# Does one size really fit all? The effectiveness of a non-diagnosis-specific integrated mental health care program in Germany in a prospective, parallel-group controlled multi-centre trial

**DOI:** 10.1186/s12888-017-1441-9

**Published:** 2017-08-01

**Authors:** Annabel Sandra Mueller-Stierlin, Marina Julia Helmbrecht, Katrin Herder, Stefanie Prinz, Nadine Rosenfeld, Julia Walendzik, Marco Holzmann, Uemmueguelsuem Dinc, Matthias Schützwohl, Thomas Becker, Reinhold Kilian

**Affiliations:** 1 Department of Psychiatry II, Ulm University, Bezirkskrankenhaus Günzburg, Ulm, Germany; 2 Institute of Epidemiology and Medical Biometry, Ulm University, Ulm, Germany; 3 Department of Psychiatry and Psychotherapy, University Hospital Carl Gustav Carus Dresden, TU Dresden, Dresden, Germany

**Keywords:** Integrated care, Assertive community treatment, Mental illness, Empowerment

## Abstract

**Background:**

The Network for Mental Health (NWpG-IC) is an integrated mental health care program implemented in 2009 by cooperation between health insurance companies and community mental health providers in Germany. Meanwhile about 10,000 patients have been enrolled. This is the first study evaluating the effectiveness of the program in comparison to standard mental health care in Germany.

**Methods:**

In a parallel-group controlled trial over 18 months conducted in five regions across Germany, a total of 260 patients enrolled in NWpG-IC and 251 patients in standard mental health care (TAU) were recruited between August 2013 and November 2014. The NWpG-IC patients had access to special services such as community-based multi-professional teams, case management, crisis intervention and family-oriented psychoeducation in addition to standard mental health care. The primary outcome empowerment (EPAS) and the secondary outcomes quality of life (WHO-QoL-BREF), satisfaction with psychiatric treatment (CSQ-8), psychosocial and clinical impairment (HoNOS) and information about mental health service needs (CAN) were measured four times at 6-month intervals. Linear mixed-effect regression models were used to estimate the main effects and interaction effects of treatment, time and primary diagnosis. Due to the non-randomised group assignment, propensity score adjustment was used to control the selection bias.

**Results:**

NWpG-IC and TAU groups did not differ with respect to most primary and secondary outcomes in our participating patients who showed a broad spectrum of psychiatric diagnoses and illness severities. However, a significant improvement in terms of patients’ satisfaction with psychiatric care and their perception of treatment participation in favour of the NWpG-IC group was found.

**Conclusions:**

Providing integrated mental health care for unspecific mentally ill target groups increases treatment participation and service satisfaction but seems not suitable to enhance the overall outcomes of mental health care in Germany. The implementation of strategies for ameliorating the needs orientation of the NWpG-IC should be considered.

**Trial registration:**

German Clinical Trial Register DRKS00005111, registered 26 July 2013.

**Electronic supplementary material:**

The online version of this article (doi:10.1186/s12888-017-1441-9) contains supplementary material, which is available to authorized users.

## Background

Comprehensive, integrated and responsive mental health and social care services provided by multi-professional teams in community-based settings are regarded by most experts as a fundamental basis of adequate contemporary mental health care [1–4]. Beyond generic community mental health teams, more intensive community-based mental health care approaches such as Home Treatment (HT) and Crisis Resolution Teams (CRT) for short-term outpatient treatment of acute psychiatric crises or Assertive Community Treatment [5] and Intensive Case Management (ICM) [6] for the long-term care of patients have been established in many countries [6–13].

In spite of the international evidence in favour of integrated care [6], in Germany ACT, ICM, HT, CRT or other intensive outreach community mental health care programmes have been rarely implemented in routine psychiatric care so far [14]. Currently psychiatric routine care in Germany is mainly provided by psychiatric hospitals, psychiatric outpatient clinics and office-based psychiatrists and psychologists. In addition, a broad spectrum of non-medical vocational, residential and psychosocial services are provided by vocational rehabilitation centres, community mental health care centres and different types of residential facilities [15]. While medical psychiatric services are mainly financed by statutory or private health insurances, vocational services are financed by unemployment funds or pension funds and psychosocial and residential services are financed by individual social benefits or taxes [15]. Due to the lack of a national mental health policy, the regional disparities in the spectrum, content and quality of services are large [15, 16]. Moreover, due to the fragmentation of service providers and payers, even within one region services are often not well integrated [16]. Particularly transition between inpatient and outpatient care is often not well coordinated and the risk of service discontinuation during this transition process is rather high [16, 17]. Health economic analyses of the German mental health care system indicate that most of the financial resources are spent for inpatient treatment and outpatient drug prescriptions while only a small part of the mental health care budget is spent for outpatient services [18–21]. It is expected that effectiveness and efficiency of the German mental health care system can be significantly improved by a shift of resources from inpatient to outpatient care [14, 22].

The legal regulations of health care financing explained above and organizational reasons build important barriers against this resource shift from inpatient to outpatient services and from medical to psychosocial services [14]. In order to reduce these barriers, a reformation of the social security code part 5 §§140a in 2004 allowed provision of integrated health care programmes including medical and psychosocial services by community mental health agencies which previously were only allowed to provide psychosocial services.

In 2009 one of the larger German health insurance companies started a model project named “Network for Mental Health” (Netzwerk für psychische Gesundheit = NWpG) that facilitates the provision of integrated mental health care services (NWpG-IC) in collaboration with local mental health service providers. The aim of NWpG-IC is to prevent psychiatric hospitalisation by provision of need-oriented community-based acute and long-term psychiatric and psychosocial care for patients with a broad spectrum of psychiatric diagnoses and mental health care needs [23].

Meanwhile, the NWpG model has been adopted by several health insurances and about 10,000 patients have been enrolled in the NWpG programme across Germany. However, the effectiveness of the program has not been systematically evaluated. In this article we present the results of an quasi-experimental prospective trial comparing the effects of integrated mental health care according to the criteria of the Network for Mental Health (NWpG-IC) to usual mental health care (TAU) in five German regions over 18 months.

## Methods

### Study design

Due to the legal and organisational framework of NWpG-IC contracts in Germany, randomisation and blinding of study participants was not feasible [24]. Therefore, a multicentre quasi-experimental prospective trial comparing outcomes of patients enrolled in the NWpG-IC programme with a control group of patients who received standard care was conducted. Preference-based group allocation was performed on the basis of type of care provision intended for the coming 18 months. The study took place in five German federal states (Schleswig-Holstein, North Rhine-Westphalia, Berlin, Saxony and Bavaria) covering a broad range of catchment areas with different levels of urbanization, with different local mental health service systems and with different providers of NWpG-IC services. Trial duration was 18 months. Data were collected at baseline and at three follow-ups after 6, 12 and 18 months.

The study was conducted in compliance with the Declaration of Helsinki 2013. It has been approved by the Ethics Committees of the University of Ulm (application number: 129/13) and of the TU Dresden (application number: EK 259072013). This trial is registered with DRKS (German Clinical Trials Register) and ICTRP (International Clinical Trials Registry Platform) with the identifier DRKS00005111. The protocol was published in 2014 [24].

### Study population and sampling procedure

Patients were eligible for enrolment in NWpG-IC contracts if they were members of one of the participating health insurances and if they fulfilled the following criteria:

- being diagnosed with a mental illness of the categories F20-F29 (schizophrenia, schizotypal and delusional disorders), F30-F39 (affective disorders), F40-F48 (neurotic, stress-related and somatoform disorders), F50-F59 (behavioural syndromes associated with physiological disturbances and physical factors), F60-F69 (disorders of adult personality and behaviour) and F91- F94 (conduct and emotional disorders, disorders of social functioning with onset usually occurring in childhood and adolescence) of the ICD-10 during the last 12 months.
- having either had a psychiatric inpatient admission or an ambulatory prescription of antipsychotic, anxiolytic or antidepressant drugs during the last 12 months.
- being between 18 and 80 years old
- not being eligible for receiving benefits from statutory long-term care insurance [25]

All criteria were assessed on the basis of information from patients. In case of implausible information the patient statements were verified by information from mental health care staff or patient records.

Patients who fulfilled eligibility criteria were offered the opportunity to participate in the NWpG-IC program by phone from their health insurances. Thereafter the patient was invited to an information meeting by the local NWpG-IC service provider. After the information meeting the patient had the opportunity to decide to enrol to NwPG-IC or not. Patients were informed that the decision for or against enrolment had no consequences for their health insurance membership status or for their reimbursement of health care expenditures.

In order to replicate the heterogeneity of the NWpG-IC target group in the study sample, a consecutive sampling procedure for two equal-sized study groups was chosen. Therefore, if a patient decided to enrol in the NWpG-IC program, he or she was asked to participate in the present study as a member of the intervention group. About one third of patients refuses to enrol in the NWpG-IC program. Those patients were asked to participate as members of the control group. In addition, control group participants were recruited in a broad spectrum of outpatient mental health care facilities (office-based psychiatrists or psychologists, outpatient clinics, day centres, residential services, community mental health care centres). Inclusion criteria were the same as for NWpG-IC eligibility (see above), with the exception of membership in one of the health insurance providers offering NWpG-IC. In order to acquire comparable study groups fulfilling the common support condition necessary for propensity score adjustment, we kept track of patients’ age, gender, employment status and ICD-10 diagnosis during recruitment. If needed the recruitment focus for the control group was adapted accordingly e.g. by trying to increase the proportion of control group participants who were employed at the time of inclusion.

### NWpG-IC intervention

In contrast to fee for service reimbursement of usual mental health care in Germany, the NWpG-IC program was financed by an annual per-capita lump sum staggered by illness severity. The lump sum was intended to cover the total costs of psychiatric hospital admissions and psychiatric outpatient treatment including additional NWpG services, but excluded costs of ambulatory medication and involuntary psychiatric admissions. Also excluded were costs of residential care which are in Germany mainly financed by taxes and costs of vocational rehabilitation which are in most part financed by unemployment or pension funds.

In contrast to TAU, the NWpG-IC service package included provision of community-based multi-professional teams, psychiatric case management, crisis intervention by means of home treatment or crisis beds in non-hospital settings, and family-oriented psychoeducation. In addition, all patients were allowed to use the available routine mental health care services such as office-based psychiatrists and psychiatric inpatient treatment.

The implementation of the programme and the budget responsibility rested with the local service providers and was independent from the research team.

### Outcomes and assessment

Beyond the alleviation of psychiatric symptoms, integrated types of community treatment commonly aim at increasing the patients’ capacities for independent living and comprehensive social and vocational inclusion [5, 6]. In order to capture this broad spectrum of outcomes comprehensively, generalized measures of quality of life, recovery and empowerment are widely used in studies related to the effectiveness of integrated care approaches [26, 27]. The primary outcome of this study was measured by means of the questionnaire for the assessment of empowerment in patients with affective and schizophrenic disorders (EPAS). The EPAS measures empowerment as the patient’s perceived opportunity to control his/her own living circumstances on five dimensions: “daily living”, “social relationships and sexuality”, “psychiatric treatment”, “hope and self-efficacy” and “self - esteem”. The EPAS core module has 33 items and has shown high reliability (Cronbach’s Alpha of 0.928) [28].

Secondary outcome measures were 1) psychosocial and clinical impairment as assessed using the Health of the Nation Outcome Scale (HoNOS) [29–32], 2) subjective Quality of Life measured by the short version of the World Health Organisation Quality of Life questionnaire (WHOQOL-BREF), 3) satisfaction with psychiatric treatment measured by using the German Version of the Client Satisfaction Questionnaire (CSQ-8) [33, 34], 4) mental health service needs as measured by the Camberwell Assessment of Needs (CAN-EU) and 5) prevalence of and number of psychiatric inpatient admissions during follow-up period estimated by the CSSRI [Client Socio-Demographic and Service Receipt Inventory] [35].

Outcome assessment was performed by trained research associates. Data and site monitoring by the study coordinator ensured quality assurance [24].

### Sample size calculation

The sample size calculation was performed for the change in the empowerment total score over 18 months. An effect size of f = 0.2 was assumed to be clinically relevant for the within-between interaction of group*time in repeated-measurements ANOVA with two groups and four time-points of measurement. Based on this effect size, a power of 0.90, and an alpha level of 0.05, a total sample size of *n* = 350 was needed. Sample size was further estimated based on a drop-out rate of 30%, according to experience from the ELAN-study [36, 37]. Based on this assumption, a total of 500 patients were recruited for visit 1. The sample size was calculated using G-Power 3.1.

### Statistical analysis

Balance between study groups was estimated by means of the absolute standardised difference. Limited balance or imbalance was indicated by absolute standardised differences greater than 0.1 or 0.2, respectively. Propensity score adjustment was used to control selection bias [38]. Propensity scores were estimated on the basis of a logistic regression model including variables that showed limited balance between study groups or that were slightly associated (*p* < 0.10) with the outcome (change in EPAS total between visit 1 and visit 4).

Linear mixed-effect regression models with a random time effect, a fixed group effect and an interaction effect between time and study group adjusted for propensity scores were computed for continuous primary and secondary outcome variables. Parametrisation of regression models occurred without group mean centering. Therefore, all effects are given with regard to the reference categories of the other model variables. The group by time interaction reflects the intervention effect. The analysis was conducted on the intention-to-treat population. Missing values were taken into account by weighting of the parameter estimation.

Logistic mixed-effect models with equivalent features were computed for the six month prevalence of psychiatric inpatient admission. The interpretation of the regression coefficients is the same as described above, with the difference that the presented odds ratios (OR) represent the proportional effects of the independent variables on the probability of the outcome event.

For investigating the effects of NWpG-IC on the number of inpatient treatment days a random-effects tobit regression model taking into account the zero inflation of the distribution was applied.

To investigate if the effect of NWpG-IC is related to the psychiatric diagnosis, we included the primary ICD-10 diagnosis group into the mixed effects model as dummy variables. The reference category was F20-F29; the three other categories were F30-F39, F40-F48 and ‘other psychiatric diagnoses’. In addition we extended the models by two-way and three-way interaction effects between treatment group, time and ICD-10 diagnosis. Due to the parameterisation of the three-way interaction models the regression coefficient for the main effect of the time variable indicates the change of the outcome variable in patients with an F20-F29 diagnosis in the control group. The main effect of the coefficient for the study group indicates the mean difference of the outcome variable between study groups in patients with an F20-F29 diagnosis at baseline and the coefficients for the main effects of the three diagnosis groups (F30-F39; F40-F48 and others) indicate the mean difference of the outcome variable between patients with F30-F39, F40-F48 or other diagnosis and patients with F20-F29 diagnosis in the control group at baseline. The coefficients for the two-way interaction effects between study group and diagnosis indicate the effect of the respective diagnosis in comparison to an F20-F29 diagnosis on the mean difference of the outcome variable between study groups at baseline. The coefficients for the two-way interaction effects between diagnosis and time indicate the effect of the respective diagnosis in comparison to an F20-F29 diagnosis on the change of the outcome variable in the control group. The coefficient for the two-way interaction effect between study group and time indicate the intervention effect of NWpG-IC in comparison to standard care in the F20-F29 diagnosis group. The coefficients for the three-way interaction between study group, diagnosis and time indicate the additional diagnosis specific intervention effect.

The repeated measurements for patients clustered according study centres were taken into account. Data capturing was performed using IBM SPSS 21. Data analysis was carried out using SAS 9.4 and STATA 14.

## Results

### Study population and study flow

**Fig. 1:**
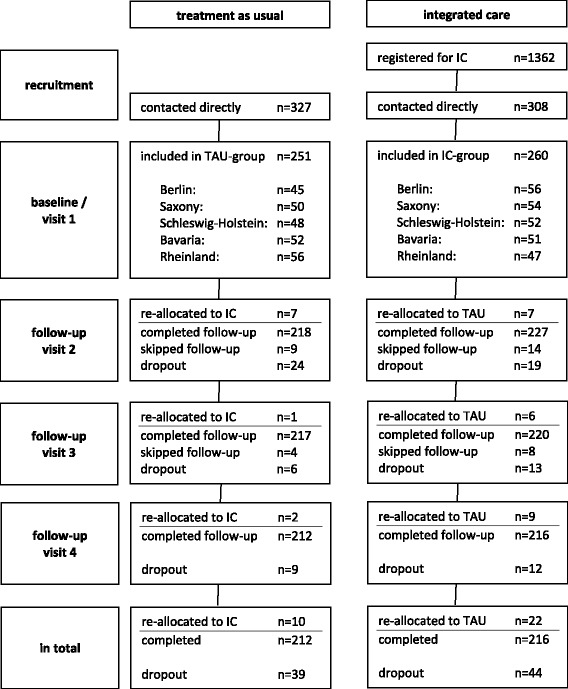
Flow of study participants. [registered for IC: number of patients that were registered for IC during recruiting phase; contacted directly: number of patients that were contacted and informed by study staff; re-allocated to IC/TAU: number of patients that re-allocated to other study group during follow-up; completed follow-up: number of patients that completed follow-up (regardless of re-allocation); skipped follow-up: number of patients that skipped follow-up, but that continued later on; dropout: number of patients that discontinued the study]

Recruitment of 511 patients (NWpG-IC = 260, TAU = 251) took place between August 2013 and November 2014. Our NWpG-IC sample comprises 19.1% of all patients that were enrolled in NWpG-IC models in the five catchment areas during the recruitment period (*N* = 1362). Altogether, 111 (21.7%) patients missed at least one assessment. In total, 428 (84.0%) patients attended the last follow-up, accordingly the dropout rate is 16%. Ten patients (4.0%) changed from the control group into the NWpG-IC program and 22 patients (8.5%) in the NWpG-IC group quitted the program after baseline assessment (Fig. 1).

As indicated in Table 1, the majority of study participants were female (353, 69.1%) and primary diagnosed with depression (317, 62.0%). The average age was 46.5 years (sd 11.6). Compared to patients in the control group those in the NWpG-IC group had a higher employment rate, a lower proportion of receiving social welfare, a shorter duration of illness, a lower number of previous inpatient admissions, and reported less needs for psychiatric and psychosocial services. In addition, a lower proportion of patients in the NWpG-IC group lived alone and had a diagnosis of schizophrenia.

**Table 1:** Sample characteristics at baseline

	Total		TAU		IC		*p*-value^a^
*Sociodemographic*							
age in years; m (sd)	46.47	11.61	47.15	11.01	45.82	12.14	0.195
employed; n (%)	175	35.1%	58	23.5%	117	46.4%	<0.001
living alone; n (%)	235	46.0%	127	50.6%	108	41.5%	0.040
female; n (%)	353	69.1%	167	66.5%	186	71.5%	0.221
health insurance company affiliation, TK; n (%)	214	41.9%	32	12.7%	182	70.0%	<0.001
social welfare reception; n (%)	26	5.1%	20	8.0%	6	2.3%	0.004
*Treatment*							
duration of illness in years; m (sd)	12.47	11.63	14.24	11.85	10.77	11.18	0.001
number of hospitalisations; m (sd)	2.78	3.89	3.83	4.92	1.77	2.1	<0.001
primary diagnosis							0.017
– F20-F29; n (%)	67	13.1%	43	17.1%	24	9.2%	
– F30-F39; n (%)	317	62.0%	140	55.8%	177	68.1%	
– F40-F48; n (%)	98	19.2%	53	21.1%	45	17.3%	
multiple mental diagnoses; n (%)	248	48.5%	135	53.8%	113	43.5%	0.020
prescription of antipsychotics; n (%)	379	74.2%	199	79.3%	180	69.2%	0.009
legal guardian; n (%)	31	6.1%	23	9.2%	8	3.1%	0.004
assisted living; n (%)	36	7.1%	31	12.4%	5	1.9%	<0.001
*Outcome measures*							
EPAS – total; m (sd)	3.42	0.60	3.42	0.62	3.42	0.59	0.959
– daily living; m (sd)	3.62	0.70	3.58	0.71	3.67	0.68	0.165
– social relationships and sexuality; m (sd)	3.21	0.72	3.20	0.75	3.23	0.70	0.640
– psychiatric treatment; m (sd)	3.66	0.66	3.72	0.67	3.61	0.64	0.045
– hope and self-efficacy; m (sd)	3.19	0.80	3.18	0.81	3.20	0.79	0.753
– self-esteem; m (sd)	3.41	0.80	3.41	0.83	3.40	0.77	0.899
– empowerment at work (*n* = 250); m (sd)	3.43	0.81	3.43	0.83	3.42	0.80	0.900
– parenting of minor children (*n* = 96); m (sd)	3.68	0.73	3.85	0.70	3.56	0.72	0.054
HONOS - total; m (sd)	10.66	5.30	10.53	5.12	10.79	5.48	0.576
number of needs; m (sd)	4.70	2.63	5.07	2.70	4.34	2.51	0.002
proportion of met needs; m (sd)	59.5%	68.2%	62.9%	69.6%	56.2%	67.2%	0.017
QOL-BREF; m (sd)	48.86	22.04	48.63	22.64	49.08	21.49	0.818
satisfaction score; m (sd)	24.22	4.43	24.51	4.53	23.94	4.33	0.154

^a^Pearson Chi^2^ test for categorical and t-test for continuous variables

Propensity score methods taking into account 31 baseline variables (see Table 2) were used to account for differences between study groups at baseline. While 16 variables were imbalanced at baseline, three variables remained imbalanced after propensity score adjustment. Those were health insurance company affiliation, proportion of unmet needs for psychiatric and psychosocial services and the number of previous psychiatric hospital admissions. The common support region of the propensity score ranges from 0.038 to 0.926 and comprises almost all study participants (*n* = 497, 97.3%).

**Table 2:** Results of the logistic regression models for the propensity score

	OR	95%-CI		
age, years [+ 1]	1.017	0.995	-	1.041
employment status^a^	1.684	1.052	-	2.695
children^a^	1.027	0.618	-	1.704
living alone^a^	0.966	0.571	-	1.637
female^a^	1.346	0.855	-	2.119
higher secondary school qualification^a^	0.752	0.432	-	1.311
in partnership^a^	0.853	0.506	-	1.439
reception of Hartz IV benefit^a^	0.858	0.458	-	1.605
reception of social welfare^a^	0.522	0.163	-	1.672
duration of illness [+ 1]	0.997	0.977	-	1.017
number of psychiatric inpatient admissions [+ 1]	1.131	1.045	-	1.225
reception of sickness benefit (last 6 months) ^a^	2.950	1.838	-	4.717
any diagnosis F20-F29^a^	1.410	0.627	-	3.175
any diagnosis F30-F39^a^	1.321	0.701	-	2.488
multiple psychiatric diagnoses^a^	0.747	0.474	-	1.176
prescription of antipsychotics^a^	0.787	0.479	-	1.294
motivation for outpatient care (instead of inpatient care) ^a^	1.845	1.149	-	2.967
mental guardian^a^	1.263	0.429	-	3.717
assisted living^a^	0.371	0.123	-	1.122
frequent contact with family^a^	1.481	0.808	-	2.717
body mass index [+1 kg/m^2^]	1.016	0.982	-	1.052
smoker^a^	0.793	0.511	-	1.230
HONOS total score [+ 1]	0.959	0.906	-	1.015
CAN needs [+ 1]	1.145	1.030	-	1.274
CAN proportion of unmet needs [+ 100%]	0.486	0.232	-	1.017
QOL-BREF total Score [+ 1]	1.006	0.991	-	1.021
QOL-dimension 1 Score [+ 1]	0.983	0.961	-	1.006
QOL-dimension 2 Score [+ 1]	1.010	0.989	-	1.031
QOL-dimension 4 Score [+ 1]	0.991	0.972	-	1.010
CSQ-8 satisfaction score [+ 1]	1.045	0.992	-	1.101
total costs (last 6 months) [+ 1 €]	1.000	1.000	-	1.000

^a^“no” as reference category of binary covariates

excluded from analysis: health insurance affiliation, reception of unemployment benefit, psychiatric inpatient admission (last 12 months), outpatient clinic, frequent contact with friends, diagnosis of a somatic illness, QOL-dimension 3 score

### Primary outcome effect

Mixed-models for EPAS total score (see Table 3) indicated a significant effect of time (b = 0.07, *p* < 0.001), but no significant group by time interaction effect (b = 0.02; *p* = 0.258). This means with regard to the parameterisation of the mixed effects model that there was a significant change of overall empowerment in the control group, but NWpG-IC treatment had no additional effect on improvement of overall empowerment during the 18 month study period.

**Table 3:** Results of mixed-effects regression models for primary and secondary outcomes

		b	se	*p*	95%-CI			n
EPAS - total	Intercept	3.27	0.06	**<0.001**	3.15	-	3.39	511
	IC	−0.10	0.07	0.406	−0.20	-	0.07	
	Time	0.07	0.01	**<0.001**	0.05	-	0.09	
	IC x Time	0.02	0.01	0.258	0.00	-	0.04	
	PS	0.23	0.12	0.054	0.00	-	0.47	
EPAS - daily living	Intercept	3.33	0.07	**<0.001**	3.20	-	3.47	511
	IC	0.00	0.08	0.934	−0.20	-	0.14	
	Time	0.06	0.01	**<0.001**	0.04	-	0.09	
	IC x Time	0.00	0.02	0.91	0.00	-	0.03	
	PS	0.44	0.13	**<0.001**	0.18	-	0.69	
EPAS - social relationships and sexuality	Intercept	3.03	0.07	**<0.001**	2.89	-	3.17	511
	IC	0.00	0.08	0.838	−0.20	-	0.14	
	Time	0.07	0.01	**<0.001**	0.05	-	0.09	
	IC x Time	0.00	0.02	0.725	0.00	-	0.03	
	PS	0.26	0.14	0.061	0.00	-	0.53	
EPAS - psychiatric treatment participation	Intercept	3.71	0.07	**<0.001**	3.58	-	3.85	511
	IC	−0.20	0.07	**0.026**	−0.30	-	0.00	
	Time	0.04	0.01	**0.005**	0.01	-	0.06	
	IC x Time	0.06	0.02	**0.001**	0.02	-	0.09	
	PS	0.01	0.13	0.928	−0.20	-	0.26	
EPAS - hope and self-efficacy	Intercept	3.02	0.08	**<0.001**	2.86	-	3.18	511
	IC	0.00	0.09	0.751	−0.20	-	0.14	
	Time	0.10	0.01	**<0.001**	0.07	-	0.13	
	IC x Time	0.01	0.02	0.698	0.00	-	0.05	
	PS	0.19	0.15	0.227	−0.10	-	0.49	
EPAS - self-esteem	Intercept	3.23	0.08	**<0.001**	3.07	-	3.39	511
	IC	−0.10	0.09	0.417	−0.20	-	0.10	
	Time	0.07	0.01	**<0.001**	0.05	-	0.10	
	IC x Time	0.02	0.02	0.352	0.00	-	0.05	
	PS	0.31	0.15	**0.045**	0.01	-	0.61	
EPAS - empowerment at work	Intercept	3.53	0.12	**<0.001**	3.30	-	3.76	358
	IC	0.12	0.13	0.328	−0.10	-	0.37	
	Time	0.09	0.03	**<0.001**	0.04	-	0.14	
	IC x Time	0.01	0.03	0.826	−0.10	-	0.07	
	PS	−0.40	0.19	**0.046**	−0.80	-	0.00	
EPAS - parenting of minor children	Intercept	3.80	0.18	**<0.001**	3.45	-	4.16	122
	IC	−0.20	0.18	0.299	−0.50	-	0.17	
	Time	0.00	0.04	0.991	−0.10	-	0.07	
	IC x Time	0.06	0.05	0.194	0.00	-	0.15	
	PS	−0.10	0.30	0.875	−0.60	-	0.55	
HoNOS - total	Intercept	11.40	0.54	**<.001**	10.30	-	12.40	511
	IC	0.74	0.58	0.2	−0.40	-	1.88	
	Time	−0.30	0.11	**0.013**	−0.50	-	−0.10	
	IC x Time	−0.30	0.16	0.108	−0.60	-	0.06	
	PS	−1.80	0.97	0.071	−3.70	-	0.15	
CAN - needs	Intercept	6.61	0.25	**<.001**	6.11	-	7.10	511
	IC	0.00	0.28	0.996	−0.60	-	0.55	
	Time	−0.50	0.05	**<.001**	−0.60	-	−0.40	
	IC x Time	0.09	0.07	0.217	−0.10	-	0.23	
	PS	−3.00	0.44	**<.001**	−3.80	-	−2.10	
CAN - proportion of met needs	Intercept	0.60	0.03	**<.001**	0.55	-	0.66	511
	IC	0.00	0.04	0.329	−0.10	-	0.03	
	Time	0.05	0.01	**<.001**	0.03	-	0.07	
	IC x Time	0.01	0.01	0.621	0.00	-	0.03	
	PS	−0.10	0.05	0.314	−0.10	-	0.04	
CSQ-8 satisfaction score	Intercept	24.80	0.43	**<.001**	23.90	-	25.60	511
	IC	−0.30	0.47	0.467	−1.30	-	0.58	
	Time	0.42	0.09	**<.001**	0.24	-	0.61	
	IC x Time	0.27	0.13	**0.039**	0.01	-	0.53	
	PS	−1.10	0.76	0.136	−2.60	-	0.36	

IC = mean difference between TAU and NWpG-IC group at baseline

Time = linear change from t0 to t3 in TAU group

IC x Time = difference in linear change between TAU and NWpG-IC group

PS = estimated coefficient for propensity-score adjustment

No entry in boldface without significance (*p* < 0.05) was identified.

### Secondary outcome effects

With exception of the EPAS dimension “psychiatric treatment participation”, no significant intervention effects for the EPAS subscales were found. For the EPAS subscale “psychiatric treatment participation” the significant effect of group (b = −0.20; *p* = 0.026) reveals that patients in the NWpG-IC group had a lower baseline value than patients in the control group. The significant time effect (b = 0.04; *p* = 0.005) suggests that the level of subjective treatment participation in the control group increased during the study period and the significant group by time interaction effect (b = 0.06; *p* = 0.001) suggests that the linear increase of subjective treatment participation was greater in the NWpG-IC group than in the control group.

The regression parameters for the HoNOS total score indicate that there was no difference in the baseline value between groups (b = 0.74; *p* = 0.200) and that the HoNOS value in the control group decreased significantly during the observation period (b = −0.30; *p* = 0.013). The non-significant group by time interaction effect (b = −0.30; *p* = 0.108) reveals that the HoNOS improvement did not differ between study groups.

The same effect patterns were found for the needs for psychiatric care. Both groups started with the same level of total needs (b = 0.28; *p* = 0.996) and the same level of met needs (b = −0.01; *p* = 0.329). The total needs significantly decreased (b = −0.50; *p* < 0.001), while the level of met needs significantly increased during the study period (b = 0.05; *p* < 0.001). However, as indicated by the non-significant group by time interaction, there was no difference between groups regarding the reduction of total needs (b = 0.09; *p* = 0.217) or the increase of met needs (b = 0.01; *p* = 0.621).

Regression parameters for treatment satisfaction point out the same level of satisfaction in both groups at baseline (b = −0.30; *p* = 0.467) and a significant increase during the study period in the control group (b = 0.42; *p* < 0.001). As indicated by the significant group by time interaction the increase of treatment satisfaction was significantly larger in the NWpG-IC group than in the control group (b = 0.27; *p* = 0.039).

The results of the mixed effects logistic regression model (not presented) did neither show differences of the prevalence of psychiatric inpatient admission between groups during the six months before baseline assessment (OR 0.99; *p* = 0.985), nor a change over time (OR 0.62; *p* = 0.086) in either groups or a difference between the groups (OR 0.97; *p* = 0.878) during the study period.

Results of the random-effects tobit regression model (not presented) indicate neither any changes of the mean number of inpatient days during the study period nor any differences between study groups.

Results of the mixed effects models including the main effect of diagnosis as well as the two-way and three-way interaction effects between study group, time and diagnosis show the following (see Additional file 1 for details): For the empowerment total score, the significant negative main effect of diagnosis group reveals that in comparison to other diagnosis groups patients with schizophrenia in the control group had a significantly higher empowerment value at baseline, but as indicated by the significant interaction effect between time and diagnosis the empowerment value in schizophrenia patients receiving TAU increased significantly less during the study period. The non-significant three-way interaction effects between group, time and diagnosis suggests that the diagnosis-specific change of the empowerment total score was not affected by the treatment group. For the subscales of the empowerment scale similar regression parameter patterns occurred.

Analogously, the regression parameters for the HoNOS total score indicate in patients with schizophrenia lower clinical and psychosocial impairment at baseline, but also a smaller amount of decrease of impairment over the course of the study.

For the proportion of met mental health service needs in relation to total needs, the regression parameters reveal that patients with F30-F39 or F40-F48 diagnoses, compared to patients with F20-F29 diagnosis, started with a lower proportion of met needs but showed a stronger increase of met needs during the study.

## Discussion

This is the first study to evaluate the effectiveness of the integrated mental health care program according to the Network for Mental Health (NWpG-IC) regarding the improvement of patients’ empowerment in a controlled multi-centre design across different regions in Germany.

The study, including 511 participants in two groups of approximately equal size, is characterized by a good data quality measured in terms of a low dropout rate and a low number of missing values. Overall, we found no general superiority of NWpG-IC over TAU in our primary and secondary outcomes. However, the study results revealed a significant greater improvement in terms of patients’ satisfaction with psychiatric care and their perception of treatment participation in favour of the NWpG-IC group.

Our study results are partly in accordance with results of a recent review on the effectiveness of ICM in comparison to standard mental health care or non-ICM for people with severe mental illness (SMI), showing that there is no evidence from randomized controlled trials for an overall improvement in mental state, social functioning, quality of life or a general reduction of relapse. But there is strong international evidence for a significant positive effect of ICM on client satisfaction [6]. In contrast to our results, the review found ICM to reduce the number of inpatient days per month when compared with standard care [6].

In one of the few German studies, Lambert et al. found better clinical outcomes as well as a reduction of psychiatric inpatient days in a group of patients with schizophrenia treated by an ACT-based integrated care program in comparison to standard care over 12 months [39]. Also in contrast to our results, Kästner et al. found improved clinical outcomes but no reduction of psychiatric hospital admissions in a group of patients with schizophrenia treated with an assertive outreach program in comparison to standard treatment over 12 months in Lower Saxony [40].

While both previous German studies included exclusively patients with schizophrenia, only about 15% of our sample had a diagnosis of schizophrenia. In addition, the severity of illness in our study sample was rather low, indicated by an average HoNOS baseline score of 11, and only one third of participating NWpG-IC patients fulfilling the criteria of severe mental disorder (at least one severe/very severe problem or at least two rather severe problems in HoNOS item 1 to 4 or 6 to 10 [41]).

Though the heterogeneity of our study sample with regard to diagnosis and illness severity is likely to reflect the target group of the NWpG-IC program [23], this heterogeneity could be a reason for the difference in results compared to other studies, particularly regarding the reduction of symptoms and of the number of psychiatric hospital admissions.

As result of the diagnosis specific subgroup analyses we found that patients with schizophrenia started at a better level of empowerment and lower level of clinical and psychosocial impairment but improved less in comparison to the other diagnostic groups. These results suggest that our sample procedure resulted not only in the selection of less severely ill patients but that this selection effect was even stronger in the group of patients with schizophrenia than in other diagnostic groups. Moreover, the lower improvement of empowerment, clinical and psychosocial impairment and met service needs in schizophrenic patients in the control group in combination with the lack of a significant interaction effect of the study group suggests, that the known weakness of usual mental health care regarding the treatment of schizophrenia [42] could not be overcome by the NWpG-IC program.

The enrolment policy of the NWpG-IC program may reflect a “one size fits all” philosophy expecting that the availability of an omnibus service package for a broad spectrum of mentally ill patients would lead to a more need-oriented provision of mental health care. However, it seems that the enrolment criteria and procedure resulted in a selection of patients who, in their majority, either did not require or were unable to make full use of the NWpG-IC service. Consequently, the potential effects of the NWpG-IC program might have been diluted to a non-detectable degree.

### Strengths and limitations

The strengths of the current study are the large sample size, which provides high statistical power. Low rates of dropout cases and missing values result in very sound data quality. Further strengths are the considerable long study duration of 18 months and the high-quality assessment of outcome criteria, which represent core concepts of contemporary mental health care targets.

The most important limitation of this study is the non-randomised assignment of study participants. Despite the application of propensity score adjustment controlling for a broad spectrum of confounding variables, we cannot exclude the possibility of a selection bias in our results. A further limitation results from the poor definition of the target group of the intervention program so that the majority of participating NWpG-IC patients was less severe ill than previously expected [24].

## Conclusions

Our study results suggest that the NWpG-IC program has the potential to increase treatment satisfaction and patients’ perceived treatment participation in patients with a broad spectrum of psychiatric diagnoses and illness severities. The lack of general effects on empowerment, clinical and psychosocial impairment, met service needs and quality of life portend that unspecific integrated mental health care programs might not be suitable to fix known shortcomings of the German mental health care system to a degree leading to a general improvement of clinical as well as psychosocial outcomes. Instead offering integrated mental health care packages to unspecific patient groups with a large variety of medical and psychosocial service needs, it seems advisable to implement strategies to improve the needs orientation of the NWpG-IC program by specifying target groups and providing need-adapted or stepped-care service packages.

## Additional file

Additional file 1: Results of logistic mixed-effects regression models including diagnosis group. (XLSX 22 kb)

## Abbreviations

CAN: Camberwell Assessment of Needs
CRT: Crisis Resolution Team
CSQ-8: German Version of the Client Satisfaction Questionnaire
CSSRI: Client Socio-Demographic and Service Receipt Inventory
EPAS: Questionnaire for the assessment of empowerment in patients with affective and schizophrenic disorders
HoNOS: Health of the Nation Outcome Scale
HT: Home treatment
IC: Integrated care
ICM: Intensive Case Management
NWpC-IC: Integrated care according to NWpG
NWpG: Netzwerk für psychische Gesundheit / Network for Mental Health
OR: Odds ratio
sd: Standard deviation
SMI: Severe mental illness
TAU: Treatment as usual
WHO-QoL-BREF: Short version of the World Health Organisation Quality of Life questionnaire
